# Ethanol Extract of *Centipeda minima* Exerts Antioxidant and Neuroprotective Effects via Activation of the Nrf2 Signaling Pathway

**DOI:** 10.1155/2019/9421037

**Published:** 2019-04-03

**Authors:** Yi-Jie Wang, Xin-Yue Wang, Xu-Yi Hao, Yong-Ming Yan, Ming Hong, Su-Fen Wei, Yi-Le Zhou, Qi Wang, Yong-Xian Cheng, Yong-Qiang Liu

**Affiliations:** ^1^Institute of Clinical Pharmacology, Guangzhou University of Chinese Medicine, Guangzhou 510405, China; ^2^Sun Yat-sen University Cancer Center, Guangzhou 510060, China; ^3^School of Pharmaceutical Sciences, Shenzhen University Health Science Center, Shenzhen 518060, China

## Abstract

Oxidative stress is implicated in the pathogenesis of neurodegeneration and other aging-related diseases. Previous studies have found that the whole herb of *Centipeda minima* has remarkable antioxidant activities. However, there have been no reports on the neuroprotective effects of *C. minima*, and the underlying mechanism of its antioxidant properties is unclear. Here, we examined the underlying mechanism of the antioxidant activities of the ethanol extract of *C. minima* (ECM) both *in vivo* and in *vitro* and found that ECM treatment attenuated glutamate and tert-butyl hydroperoxide (tBHP)-induced neuronal death, reactive oxygen species (ROS) production, and mitochondria dysfunction. tBHP-induced phosphorylation of p38 mitogen-activated protein kinase (p38 MAPK) and c-Jun N-terminal kinases (JNK) was reduced by ECM, and ECM sustained phosphorylation level of extracellular signal regulated kinase (ERK) in SH-SY5Y and PC12 cells. Moreover, ECM induced the activation of nuclear factor erythroid 2-related factor 2 (Nrf2) and the upregulation of phase II detoxification enzymes, including heme oxygenase-1 (HO-1), superoxide dismutase-2 (SOD2), and NAD(P)H quinone oxidoreductase-1 (NQO-1) in both two cell types. In a D-galactose (D-gal) and aluminum muriate (AlCl_3_)-induced neurodegenerative mouse model, administration of ECM improved the learning and memory of mice in the Morris water maze test and ameliorated the effects of neurodegenerative disorders. ECM sustained the expression level of postsynaptic density 95 (PSD95) and synaptophysin (SYN), activated the Nrf2 signaling pathway, and restored the levels of cellular antioxidants in the hippocampus of mice. In addition, four sesquiterpenoids were isolated from *C. minima* to identify the bioactive components responsible for the antioxidant activity of *C. minima*; 6-*O*-angeloylplenolin and arnicolide D were found to be the active compounds responsible for the activation of the Nrf2 signaling pathway and inhibition of ROS production. Our study examined the mechanism of *C. minima* and its active components in the amelioration of oxidative stress, which holds the promise for the treatment of neurodegenerative disease.

## 1. Introduction

Aging is a complex molecular process that is associated with many life-threatening diseases, such as neurodegenerative disease, diabetes, and cardiovascular disease [1, 2]. The hippocampus is the most vulnerable region in the central nervous system (CNS), as it can be severely affected by the aging process [3]; neurodegeneration can be induced in the hippocampus and can result in cognitive dysfunction, which has a close relationship with the pathological progression of Alzheimer's disease and markedly decreases quality of life [4–6]. Various drugs have been developed to ameliorate neurodegenerative diseases. Donepezil is one of the most commonly used drugs approved for dementia; however, adverse side effects could be induced by long-term and high-dose treatment [7–9]. Development of novel drugs for the prevention and treatment of neurodegenerative diseases is urgently needed.

Oxidative damage, mitochondrial dysfunction, and carbonyl toxification have been widely accepted as the primary causes for the development of aging processes, especially the pathogenesis of most neurodegenerative disorders [10, 11]. Previous research has shown that high levels of ROS and abnormal redox changes can markedly induce neuronal death and potentiate the pathogenesis of neurodegenerative disease [12]. Excessive free radicals can attack biological macromolecules, including nucleic acids, lipids, and proteins through peroxidation, which induces malonyl dialdehyde (MDA) production, nucleic acid crosslinking, and antioxidative enzyme overconsumption, consequently leading to neurological senescence in the hippocampus and cortex [13–15]. Several signaling pathways have been reported to protect normal tissues from oxidative damage, and compelling evidence has demonstrated that the phase II detoxification systems exert neuroprotective effects against carcinogens and oxidants via the Nrf2 signaling pathways [16, 17]. Nrf2 is a pivotal regulator that can activate genes in the CNS, such as HO-1, NQO-1, SOD1, glutathione peroxidase, thioredoxins, and glutathione S-transferase (GST) [18–20]. Several studies have shown that HO-1 and NQO-1 exert neuroprotective effects by directly reducing oxidative stress and maintaining the integrity of the mitochondria [21, 22]. Meanwhile, altered levels of HO-1 and NQO-1 expression have been found in the temporal cortex and hippocampus of patients with dementia [23, 24]. Furthermore, Nrf2 overexpression has been shown to protect against neurotoxicity caused by amyloid fibrils [25, 26], indicating that phase II detoxification enzymes have an indispensable role in alleviating the pathogenesis of neurodegenerative disease. Therefore, stimulation of the Nrf2 signaling pathway could be a valuable tool for amelioration of oxidative stress and treatment of neurodegenerative diseases.

Traditional Chinese medicine (TCM) has been widely used for the treatment of aging diseases based on its antioxidant properties. The crude extracts and active components in TCM exert antioxidant activities either by directly scavenging free radical or enhancing the function of the antioxidant enzymes [27]. *C. minima* is widely distributed over the areas of East and Southeast Asia, is well known as a medicinal herb that has antibacterial and antiprotozoal activities, and is used for the treatment of nasal allergy, headache, cough, malaria, and asthma in China and Korea [28–31]. Both the aqueous and hydroalcoholic extracts of *C. minima* have been reported to attenuate oxidative stress by increasing the activities of antioxidant enzymes, which suggests that *C. minima* may contain bioactive components that have antioxidant properties [32]. Several compounds, including sesquiterpene lactones and terpenoids, have been isolated from *C. minima* and tested for antiproliferation activity in cancer cells [33–37]. However, the role of *C. minima* in treating neurodegenerative diseases remains unknown, and the bioactive components of *C. minima* and their antioxidant activities have never been reported. Here, we examined the neuroprotective effects of ECM against oxidative stress and explored its underlying mechanism both *in vivo* and *in vitro*. We also tested the antioxidant activity of the sesquiterpene lactones isolated from ECM. Our results suggest that *C. minima* and its isolated bioactive compounds hold the promise as antioxidant agents for the treatment of aging-related neurodegenerative diseases.

## 2. Materials and Methods

### 2.1. Materials

tBHP, D-gal, AlCl_3_, and glutamate were purchased from Sigma-Aldrich (St. Louis, MO). Kits used for MDA, SOD, glutathione (GSH), the bicinchoninic acid (BCA) protein assay, the ROS assay, and mitochondrial membrane potential detection were purchased from Beyotime (Shanghai, China). Antibodies against Nrf2, HO-1, SOD2, NQO-1, and Lamin B1 were obtained from Abcam (Cambridge, MA). Antibodies against *β*-actin, phospho-p38, p38, phospho-ERK, ERK, phospho-JNK, GAPDH, SYN, PSD95, Bcl-2, and Bax were purchased from Cell Signaling Technology (Beverly, MA). All secondary antibodies (horseradish peroxidase-conjugated anti-rabbit IgG and anti-mouse IgG) were purchased from Cell Signaling Technology (Beverly, MA). All reagents used were of the highest grade available commercially.

### 2.2. *Centipeda minima* Extract Preparation

The *C. minima* powders were extracted by 95% EtOH with ultrasound (twice in 6-fold solvent for 1 h each) followed by concentration *in vacuo* and lyophilization to produce a 10.3% yield dried extract. The ECM was stored at 4°C until use. For the cell treatment, the ECM sample was dissolved in DMSO to make a stock concentration of 20 mg/ml. For the animal experiments, the ECM sample was dissolved in a solution of 10% ethanol in normal saline to the concentrations of 20, 40, and 80 mg/ml, which corresponded to the low-, middle-, and high-dose groups of ECM, respectively. The compound 6-*O*-angeloylplenolin, referred to as EBSC-26A, was purified from the ethanol extract of *C. minima* as previously described [37]. We separated and enriched the EBSC-26A by high-performance liquid chromatography (HPLC) and also got the other three compounds EBSC-26B–EBSC-26D (see Supplementary Information). The structures of EBSC-26A to EBSC-26D with HPLC grade purity were identified as 6-*O*-angeloylplenolin, arnicolide D, arnicolide C, and microhelenin C by spectroscopic methods as well as comparison with literature data [28, 38] (Supplementary Figures S2–S9). The samples were dissolved in DMSO at a stock solution of 20 mmol/l.

### 2.3. Cell Culture and Treatment

The human neuroblastoma cell line SH-SY5Y was purchased from the cell bank Interlab Cell Line Collection (Genova, Italy). The highly differentiated mouse pheochromocytoma line PC12 was obtained from American Type Culture Collection (Rockville, MD). Cells were cultured in DMEM medium (Gibco, CA) contained with 10% fetal bovine serum (Gibco, CA), penicillin (100 U/ml, Gibco, CA), and streptomycin (100 mg/ml, Gibco, CA) in a humidified incubator containing 95% air and 5% CO_2_ at 37°C. For the experiments, the cells were pretreated with the indicated concentrations of ECM (0.5, 1, or 2 *μ*g/ml) or the compounds EBSC-26A−EBSC-26D (0.5, 1, or 2 *μ*M) for 2 h prior to the addition of tBHP (300 *μ*M) and glutamate (10 mM), and vitamin E was used as a positive control for investigation of antioxidant activity of ECM.

### 2.4. Animals

All experiments in this study were approved by and performed in accordance with the guidelines of the Animal Ethics Committee of Guangzhou University of Chinese Medicine. Six-week-old male Kunming mice were purchased from Shanghai Laboratory Animal Research Center (Shanghai, China). The mice were kept for a minimum of one week prior to the experiments at a temperature of 23 ± 1°C on a 12 h light/dark cycle with access to water and food *ad libitum*. The mice were randomly divided into six groups (*n* = 10 per group): (1) vehicle control (solvent: 10% ethanol in normal saline); (2) D-gal (120 mg/kg)/AlCl_3_ (20 mg/kg); (3) D − gal/AlCl_3_ + ECM (100, 200, or 400 mg/kg); (4) D-gal/AlCl_3_ + vitamin E (80 mg/kg); Vitamin E (16 mg/ml, dissolved with 10% ethanol in normal saline) was used as apositive control. The experimental groups were treated with D-gal (hypodermic injection, 120 mg/kg/d, Sigma-Aldrich, St. Louis, MO) and AlCl_3_ (intragastric administration, 20 mg/kg/d, Sigma-Aldrich, St. Louis, MO) for 90 days to establish a subacute aging model. After treatment with D-gal and AlCl_3_ for an uninterrupted 60 days, ECM and vitamin E were administrated intragastrically to each of the treatment group every day for 30 d. At the end of the treatment period, behavioral tests were performed at regular intervals to assess learning and memory of the mice.

### 2.5. Cell Viability

MTT assay was used to detect the viability of SH-SY5Y and PC12 cells in 96-well plates. 1 × 10^4^ cells per well were pretreated with ECM or EBSC-26A−EBSC-26D at the indicated concentrations for 2 h and further incubated with tBHP (300 *μ*M) for 6 h or glutamate (10 mM) for 24 h. The cell morphology was observed using a Leica optical microscope at the end of treatment. 10 *μ*l of an MTT working solution containing 5 mg/ml MTT was added into each well and incubated for 3 h at 37°C. The supernatant was then replaced with 100 *μ*l of DMSO, and the absorbance was detected at 490 nm using a microplate reader (Thermo Fisher, Waltham, MA). The results were expressed as the mean percentage of absorbance (treated versus control cells).

### 2.6. Determination of ROS Production

The intracellular ROS levels were measured using a 2′,7′-dichlorofluorescin diacetate (DCFH-DA) fluorescent probe as previously described [39]. Briefly, SH-SY5Y and PC12 cells were washed with ice-cold PBS and incubated with 5 *μ*M DCFH-DA in phenol red-free DMEM medium for 30 min at 37°C in the dark. The cells were then washed with PBS and stained with Hoechst 33342 for 5 min. DCFH-DA will be cleaved by intracellular esterases and be oxidized into the highly fluorescent dichlorofluorescein (DCF) by ROS. ROS-positive cells were monitored using a fluorescence microscope (Spectra MAX, Gemini EM, Molecular Devices, Sunnyvale, CA).

### 2.7. Measurement of MDA, GSH, and SOD Activities

The contents of the MDA and GSH, and the activities of SOD were determined as described previously [40]. Briefly, the cells were harvested and lysed in 0.2 ml of lysis buffer (1% Triton X-100 in PBS, pH 7.0) with sonication on ice. The homogenate was centrifuged at 13,200×g for 10 min at 4°C, and the supernatant was collected to determine the activity of SOD and the contents of MDA and GSH using assay kits (Beyotime, Shanghai). For the *in vivo* tests, the hippocampus and cortex were homogenized in ice-cold saline. The homogenate was centrifuged at 13,200×g for 10 min at 4°C. The supernatant was collected for the measurement of the activity of SOD and the contents of MDA and GSH according to the manufacturer's instructions. The total protein concentration of the supernatant was determined using a BCA Protein Assay.

### 2.8. Determination of Mitochondrial Membrane Potential (MMP)

The fluorescent probe JC-1 exists as a green fluorescent monomer in cells at low MMP and forms red fluorescent aggregates at high MMP and can be used to measure MMP as previously described [41]. In brief, the cell culture medium was removed and the cells were further incubated with 500 *μ*l of Hank's solution containing 10 mg/ml JC-1 for 20 min at 37°C. After removal of the Hank's solution, the cells were washed with PBS. The green fluorescence of the JC-1 monomer and the red fluorescence of the JC-1 oligomer were observed using fluorescence microscopy.

### 2.9. Extracting Cytoplasmic and Nuclear Proteins

The cultured cells were harvested and washed twice with cold PBS. The cytoplasmic and nuclear protein fractions were extracted using a Nuclear and Cytoplasmic Extraction Kit (Thermo Fisher Scientific, Rockford, IL) according to the manufacturer's protocol.

### 2.10. Morris Water Maze Test

The Morris water maze test was performed according to the method described by Morris [42]. The water maze equipment (Guangzhou Feidi Biology Technology Co. Ltd., Guangzhou, China) consists of a black circular pool, black platform, and recording system. The pool was divided into four imaginary quadrants (target, opposite, left, and right) by a computerized tracking/image analysis system. A circular, transparent escape platform (10 cm diameter) was placed 2 cm below the surface of the water in the target quadrant of the pool. The learning and memory abilities of the mice were assessed using the Morris water maze test in a dark room. The mice were given a place navigation test on five consecutive days. Each daily trial consisted of four sequential training trials that began with placing the animal in the water facing the wall of the pool. The drop location changed for each trial at random. The recording system started to record the time upon placement of the animal in the water. The escape latency was recorded at the time required for the mice to find the platform. If the mice failed to find the platform within 90 s, it would be guided to the platform by the trainer and was allowed to remain there for 10 s; in this instance, the escape latency was recorded as 90 s. On the sixth day, the mice were allowed to swim freely in the pool for 90 s without the platform, and the number of crossing through the original platform position was recorded.

### 2.11. Western Blotting

At the end of treatment, the cells and tissues were harvested and lysed using RIPA lysis buffer. Equal amounts of protein per sample were loaded in each lane and separated by 12% SDS-PAGE and then transferred to PVDF membranes. The membranes were blocked with 5% skimmed milk for 2 h at room temperature and incubated with the indicated antibodies overnight at 4°C. The PVDF membranes were further incubated with HRP-conjugated secondary antibodies for 2 h at room temperature. The protein bands were visualized using a Super Signal West Pico Chemiluminescent Substrate Trial Kit (Pierce, Rockford, IL). Images were obtained using a ChemiDoc XRS system with Quantity One software (Bio-Rad, Richmond, CA). The results were obtained from a minimum of three independent experiments.

### 2.12. Statistical Analyses

The data are presented as the mean ± SD unless noted otherwise. Unpaired *t*-tests or one-way analysis of variance (ANOVA) followed by Dunnett's post hoc tests were performed to determine the statistical significance of the differences. *P* < 0.05 was considered statistically significant (error bars, SEM). Data handling and statistical processing were performed using GraphPad Prism 7.0 (GraphPad Software, San Diego, CA). All experiments were performed at least three times.

## 3. Results

### 3.1. ECM Attenuates Oxidative Stress-Induced Cell Death

**Figure 1:**
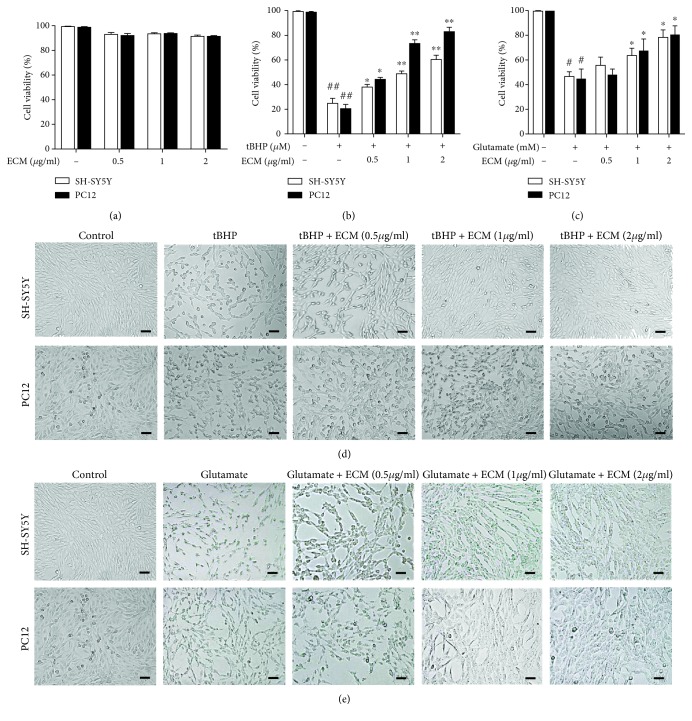
ECM inhibits oxidative stress-induced cell death. (a) Relative viability of SH-SY5Y and PC12 cells treated with 0.5-2 *μ*g/ml ECM at 37°C for 24 h. (b) Relative viability of SH-SY5Y and PC12 cells pretreated with 0.5-2 *μ*g/ml ECM for 2 h and then exposed to 300 *μ*M tBHP for an additional 6 h. (c) Relative viability of SH-SY5Y and PC12 cells pretreated with 0.5-2 *μ*g/ml ECM for 2 h and then exposed to 10 mM glutamate for an additional 24 h. Cells were pretreated with ECM and then treated with 300 *μ*M tBHP for 4 h (d) or 10 mM glutamate for 18 h (e). The morphological changes were observed in the bright field of microscope. Scale bar, 50 *μ*m. All data are normalized to control cells and presented as the mean ± SEM of three independent experiments. ^#^ *p* < 0.05 and ^##^ *p* < 0.01 in comparison with control cells. ^∗^ *p* < 0.05 and ^∗∗^ *p* < 0.01 in comparison with the cells exposed to glutamate (b) or tBHP (c) alone.

To determine the neuroprotective effects of ECM against oxidative stress, the well-known ROS inducers tBHP and glutamate were used to generate excessive ROS in neuronal SH-SY5Y and PC12 cells. First, a cell viability assay was performed to examine the toxic effects of ECM on neuronal cells. No obvious cytotoxicity of ECM was observed at the indicated concentrations (Figure 1(a)), suggesting that ECM is safe for neuronal cells. We then tested the neuroprotective effects of ECM. tBHP and glutamate treatment reduced the cell viability of SH-SY5Y and PC12 cells to 30-50% at the indicated time points. However, ECM pretreatment protected the cells against oxidative damage, as the ratio of viable cells of SH-SY5Y and PC12 increased to nearly 90% (Figures 1(b) and 1(c)). Cell morphological changes were examined to detect the neuroprotective effects of ECM. SH-SY5Y and PC12 cells shrank and became round after treatment with tBHP and glutamate, suggesting that massive cytotoxicity had been induced by excessive oxidative stress. ECM pretreatment reversed the morphological changes, with the cells treated with ECM at a high dose almost returning to normal in comparison with the control cells (Figures 1(d) and 1(e)). These results indicated that ECM exerted neuroprotective effects through the attenuation of oxidative stress.

### 3.2. ECM Inhibits ROS Production and Mitochondria Dysfunction in Neuronal Cells

**Figure 2:**
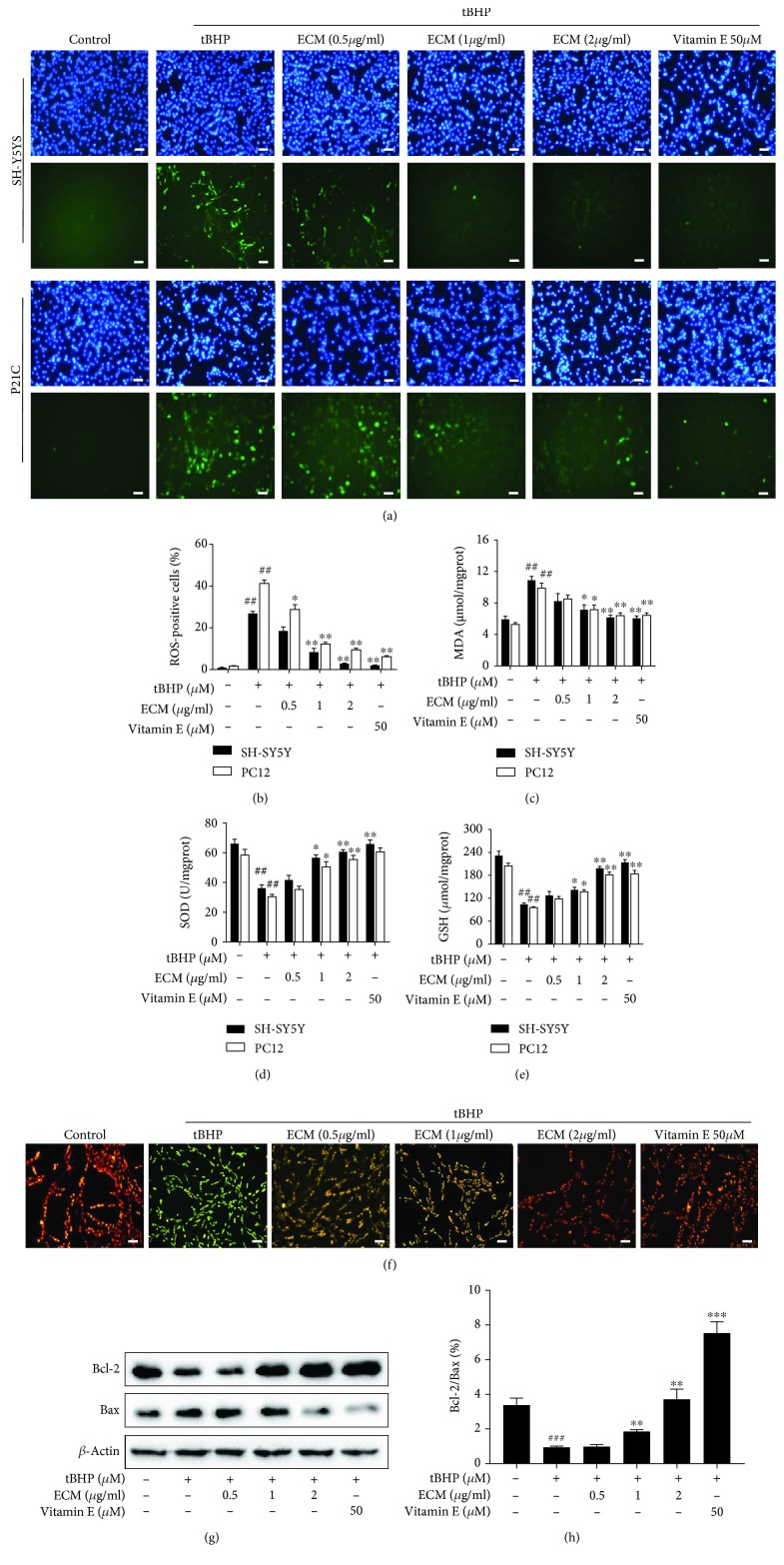
ECM attenuates oxidative stress-induced mitochondrial dysfunction. The cells were pretreated with 0.5-2 *μ*g/ml ECM and 50 *μ*M vitamin E for 2 h and then exposed to 300 *μ*M tBHP for an additional 6 h. (a) SH-SY5Y (upper panel) or PC12 cells (lower panel) were treated with DCFH-DA for 30 min; Hoechst 33342 was used to counterstain cell nuclei. Scale bar, 50 *μ*m. (b) The percentage of ROS-positive cells among cultured SH-SY5Y or PC12 cells was quantified and was shown as histogram. Intracellular MDA content (c), SOD activity (d), and GSH levels (e) were detected using a kit assay and are presented as a histogram. (f) SH-SY5Y cells were pretreated with 0.5-2 *μ*g/ml ECM for 2 h and then exposed to 300 *μ*M tBHP for an additional 6 h. The mitochondrial membrane potential was determined using the JC-1 fluorescence probe, and representative pictures have been shown for comparison. (g) Western blot analysis was performed using antibodies against Bax and Bcl-2, and *β*-actin was used as a loading control. (h) The ratio of Bcl-2 to Bax was quantified by densitometry and is shown as a histogram. The results are shown as the mean ± SEM of three independent experiments. ^##^ *p* < 0.01 in comparison with control cells. ^∗^ *p* < 0.05 and ^∗∗^ *p* < 0.01 in comparison with the cells exposed to tBHP alone.

To confirm the neuroprotective effect of ECM via the amelioration of oxidative stress, we detected the ROS scavenging activity of ECM in SH-SY5Y and PC12 cells using the oxidation-sensitive fluorescent probe DCFH-DA. The results showed that tBHP significantly increased intracellular ROS generation in SH-SY5Y and PC12 cells relative to the control cells that the effect could be reversed by ECM and vitamin E treatment, and that ECM treatment at a high dose had a ROS scavenging activity comparable to vitamin E (Figures 2(a) and 2(b)). Moreover, MDA production was increased, and the levels of GSH and SOD activities were decreased after tBHP treatment (Figures 2(c) and 2(e)), indicating that intracellular antioxidative capacity had been reduced. ECM and vitamin E pretreatment significantly decreased the level of MDA and increased the level of GSH and SOD activities in SH-SY5Y and PC12 cells (Figures 2(c) and 2(e)).

Excessive ROS generation can induce depolarization of mitochondria and changes in the MMP, which accelerate ROS-induced neuronal damage. Moreover, Bcl-2 family proteins are major regulators of MMP, Bcl-2 possesses antiapoptotic activity, whereas Bax exerts proapoptotic effect, and the Bcl-2/Bax ratio is of particular interest in assessing mitochondria-mediated cell death. To determine the role of ECM in mitochondrial protection, we detected MMP by JC-1 staining and further examined the ratio of Bcl-2/Bax expression by Western blot. The control cells with normal MMP exhibited red fluorescence after JC1 staining; however, tBHP treatment for 6 h significantly increased the ratio of green/red fluorescence (Figure 2(f)), representing a decline in MMP in the SH-SY5Y cells. Meanwhile, ECM and vitamin E treatment markedly attenuated the tBHP-induced collapse of MMP, as indicated by an increased red/green florescence ratio (Figure 2(f)). Consistently, exposure to tBHP decreased the ratio of Bcl-2/Bax expression in comparison with control cells, whereas ECM treatment increased the ratio of Bcl-2/Bax (Figures 2(g) and 2(h)), suggesting that ECM sustained MMP and protected mitochondrial function from oxidant-induced damage, thus exerting neuroprotective effects.

### 3.3. ECM Attenuates tBHP-Induced Oxidative Stress through Modulation of the MAPK and Nrf2 Signaling Pathways

**Figure 3:**
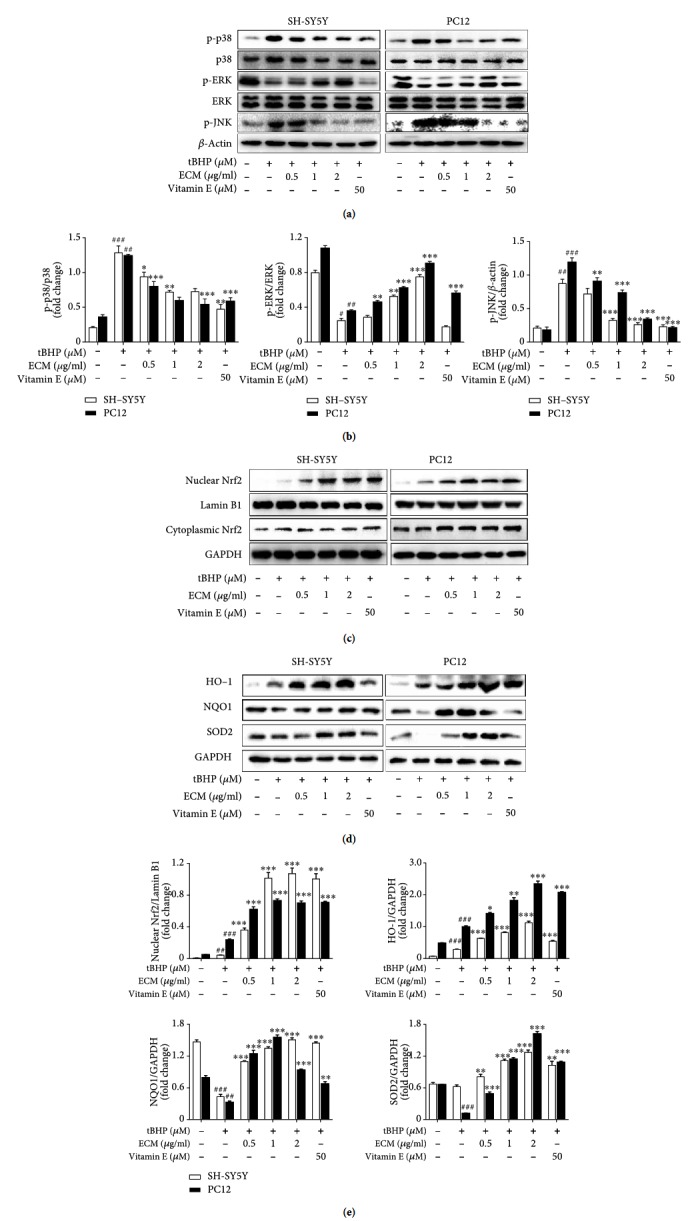
ECM inhibits oxidative stress via the regulation of the MAPK and Nrf2 signaling pathways. The cells were pretreated with 0.5-2 *μ*g/ml ECM and 50 *μ*M vitamin E for 2 h and then exposed to 300 *μ*M tBHP for an additional 1 h. Western blot analysis was performed using antibodies against p-p38, p38, p-ERK, ERK, p-JNK, and GAPDH; *β*-actin was used as a loading control in SH-SY5Y (a) and PC12 cells (b). Nuclear and cytoplasmic proteins were extracted after treatment, and Nrf2 was detected using Western blot analysis in SH-SY5Y (c) and PC12 cells (d); GAPDH and Lamin B1 were used as loading controls. Western blot analysis was performed using antibodies against HO-1, NQO1, and SOD2 in SH-SY5Y (e) and PC12 cells (f); GAPDH was used as a loading control.

It has previously been demonstrated that oxidative stress can activate members of MAPK family such as p38 MAPK and JNK, which are critical in mediating intracellular stress. We further assessed whether ECM exerts antioxidant effects via regulation of the MAPK kinase pathway. We found that tBHP exposure for 1 h caused a significant increase in the phosphorylation levels of p38 MAPK and JNK in SH-SY5Y and PC12 cells. However, preincubation with ECM markedly attenuated the tBHP-induced p38 MAPK and JNK phosphorylation (Figures 3(a) and 3(b)). In contrast, the phosphorylation level of ERK was significantly decreased in cells exposed to tBHP, while ECM pretreatment increased the phosphorylation level of ERK (Figures 3(a) and 3(b)). These results suggest that alteration of the phosphorylation level of MAPK kinase can mediate the antioxidant activity of ECM, and that p38 MAPK and ERK may play different role in mediating the antioxidant effects of ECM.

ERK has been reported to regulate Nrf2 nuclear translocation and the antioxidative response, and numerous studies have demonstrated that natural products can improve the antioxidant capacity by increasing the level of phase II detoxification enzymes. We examined whether ECM improved the intracellular antioxidant capacity via regulation of the Nrf2 signaling pathway. SH-SY5Y and PC12 cells were pretreated with ECM for 2 h before exposing them to tBHP for an additional 6 h. We found that ECM treatment obviously increased the nuclear level of Nrf2 in the presence of tBHP, while the cytoplasmic part of Nrf2 did not change (Figures 3(c) and 3(e)), suggesting that ECM inhibited Nrf2 degradation and induced Nrf2 nuclear translocation. Further, the expression levels of the Nrf2 downstream proteins, the phase II detoxification enzymes, were checked by Western blot. tBHP treatment alone slightly altered the expression levels of HO-1, SOD2, and NQO-1. However, pretreatment with ECM significantly upregulated the expression levels of HO-1, SOD2, and NQO-1 in the presence of tBHP (Figures 3(d) and 3(e)). Our results indicated that ECM has a role in attenuating ROS production via the enhancement of intracellular antioxidant capacity.

### 3.4. Active Compounds in ECM Alleviate tBHP-Induced Oxidative Stress through the Nrf2 Signaling Pathway

**Figure 4:**
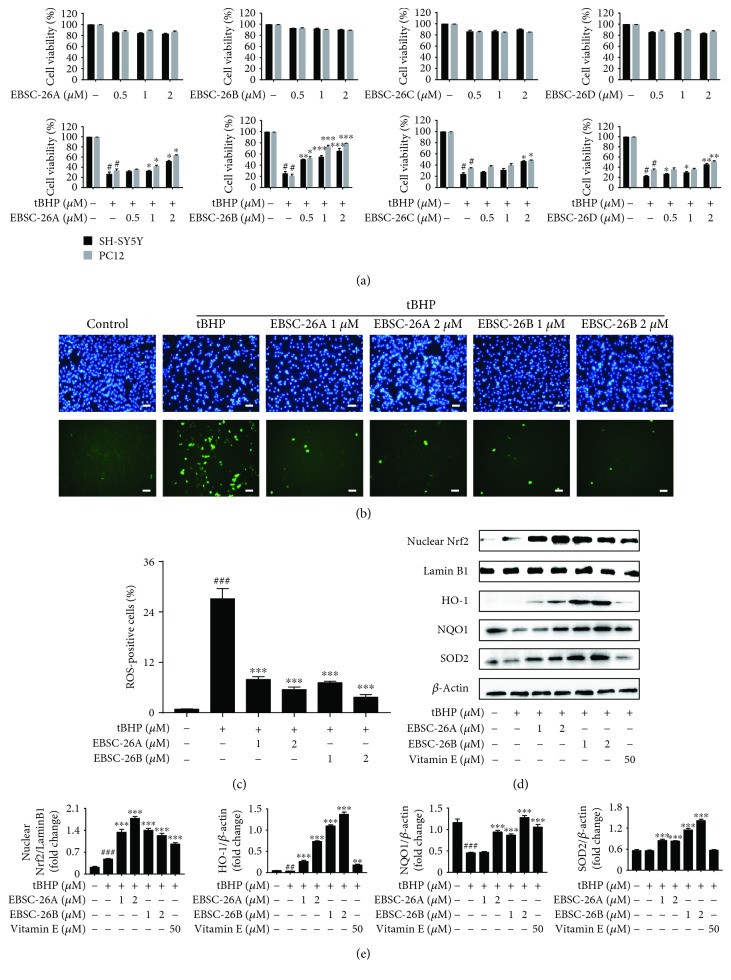
The active compounds EBSC-26A−EBSC-26D ameliorate oxidative stress. (a) SH-SY5Y and PC12 cells were treated with 0.5-2 *μ*M EBSC-26A−EBSC-26D at 37°C for 24 h (upper panel) or pretreated with 0.5-2 *μ*M EBSC-26A−EBSC-26D for 2 h and then exposed to 300 *μ*M tBHP for an additional 6 h (lower panel); the relative viability of cells was measured by MTT assay. (b, c) SH-SY5Y cells were probed with DCFH-DA (b) and the percentage of ROS-positive cells among culture cells was quantified (c). Scale bar, 50 *μ*m. (d) After pretreatment with 1-2 *μ*M EBSC-26A, EBSC-26B, and vitamin E for 2 h, SH-SY5Y cells were exposed to 300 *μ*M tBHP for an additional 6 h, and then nuclear Nrf2, HO-1, NQO-1, and SOD2 levels were detected by Western blot; *β*-actin was used as a loading control. (e) Relative protein levels were quantified by densitometry and normalized to Lamin B or *β*-actin. The results are shown as the mean ± SEM of three independent experiments. ^#^ *p* < 0.05 in comparison with control cells. ^∗^ *p* < 0.05 and ^∗∗^ *p* < 0.01 in comparison with the cells exposed to tBHP alone.

Recently, several sesquiterpene lactones have been isolated from *C. minima*. However, the active compounds that contribute to the antioxidative properties of *C. minima* have never been reported. In order to identify the active components that exert neuroprotective effects, we isolated a series of sesquiterpenoids from the ethanol extracts of *C. minima*, including 6-*O*-angeloylplenolin, arnicolide D, arnicolide C, and microhelenin C, referred to as EBSC-26A−EBSC-26D, respectively. We first tested the antioxidative activities of these compounds. None of the compounds showed obvious cytotoxicity towards SH-SY5Y or PC12 cells at indicated concentrations (Figure 4(a), upper panel). Intriguingly, the compounds protected the neuronal cells from the oxidative damage induced by tBHP (Figure 4(a), lower panel). In particular, EBSC-26A and EBSC-26B significantly reverse the cytotoxic effects induced by tBHP at lower-dose treatment. Meanwhile, the tBHP-induced ROS production was also attenuated by EBSC-26A and EBSC-26B (Figures 4(b) and 4(c)). We then examined whether EBSC-26A and EBSC-26B exerted antioxidant effects via the Nrf2 signaling pathway. SH-SY5Y cells were pretreated with EBSC-26A and EBSC-26B for 2 h before tBHP treatment for an additional 6 h. We found that both EBSC-26A and EBSC-26B markedly increased the nuclear levels of Nrf2 and subsequently upregulated the expression levels of HO-1, NQO-1, and SOD2 (Figures 4(d) and 4(e)), suggesting that the sesquiterpenoids in *C. minima* act as the active compounds that attenuate oxidative stress, and that 6-*O*-angeloylplenolin and arnicolide D are the potent antioxidant compounds in *C. minima*.

### 3.5. ECM Improves Learning and Memory Ability in a D-gal/AlCl_3_-Induced Mouse Model

**Figure 5:**
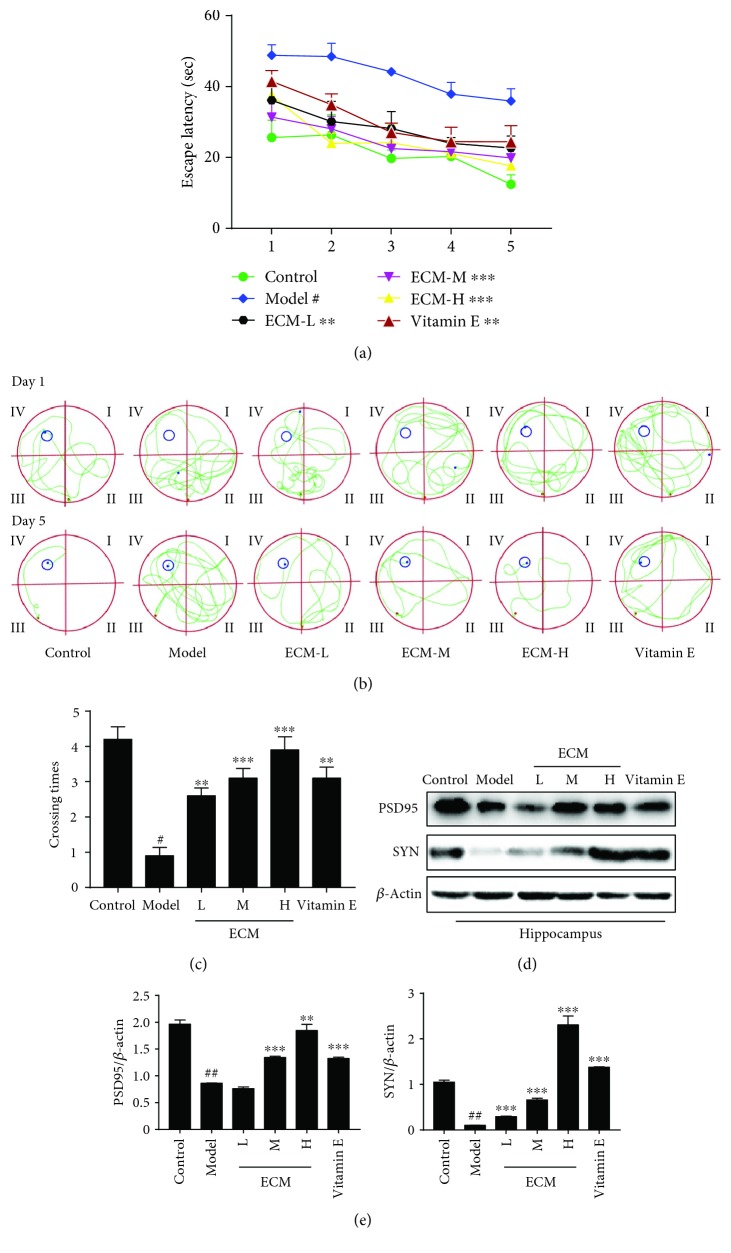
ECM improves learning and memory in a neurodegenerative mouse model. (a) Escape latency on five consecutive days of testing. (b) The swimming paths of the respective groups on the first and fifth days. (c) The crossing times in the probe trial. (d) Western blot analysis of PSD95 and SYN; *β*-actin was used as a loading control in the hippocampus. (e) Relative protein levels were quantified by densitometry and normalized to *β*-actin. Control: vehicle control; ECM-L: D − gal/AlCl_3_ + ECM (100 mg/kg/d); ECM-M: D − gal/AlCl_3_ + ECM (200 mg/kg/d); ECM-H: D − gal/AlCl_3_ + ECM (400 mg/kg/d); vitamin E: D − gal/AlCl_3_ + vitamin E (80 mg/kg/d). Data represent mean ± SEM (*n* = 10 per group). ^#^ *p* < 0.05 and ^##^ *p* < 0.01 in comparison with control group; ^∗^ *p* < 0.05, ^∗∗^ *p* < 0.01, and ^∗∗∗^ *p* < 0.001 in comparison with the model group.

D-gal can produce oxidative stress and induce neurodegeneration and memory impairment. AlCl_3_ is also frequently used to reduce neuronal viability in the hippocampus and promote memory impairment. Thus, we evaluated the therapeutic potential of ECM in a D-gal/AlCl_3_-induced neurodegenerative mouse model. The mice were treated with D-gal/AlCl_3_ for 3 months to induce neurodegeneration. ECM was administrated during the final month of D-gal/AlCl_3_ treatment, and the Morris water maze test was performed to assess learning and memory. During the five days of spatial acquisition training, we found that the time required for the mice to find the hidden platform decreased progressively (Figure 5(a)). The D-gal/AlCl_3_ group exhibited significantly longer escape latency than the vehicle control group. ECM or vitamin E treatment decreased the escape latency (Figure 5(a)). The ECM or vitamin E treatment group had a shorter swimming path to the platform than the D-gal/AlCl_3_ group (Figure 5(b)). Subsequently, the platform was removed on day 6, the spatial probe test was performed, and the number of crossings over the position of the removed platform was recorded (Figure 5(c)). The ECM or vitamin E treatment group had significantly more platform crossings than the mice in the D-gal/AlCl_3_ group (Figure 5(c)), indicating that ECM could improve learning and memory.

The synaptic proteins, PSD95 and SYN, play critical roles in synaptic plasticity and cognitive function [43, 44]. Oxidative stress can induce a decrease of the expression of PSD95 and SYN in the hippocampus, leading to cognitive impairment and development of neurodegenerative diseases [45, 46]. Thus, we examined the neuroprotective effects of ECM on the hippocampus. In D-gal/AlCl_3_-treated mice, we found that the expression levels of PSD95 and SYN were decreased in the hippocampal tissues (Figure 5(d)); meanwhile, the neurons in the hippocampus were remarkably shrunken and irregularly arranged, and the pyknotic nuclei were also increased based on H&E staining (Supplementary Figure 1), indicating that oxidative stress contributes to neuronal damage in the hippocampus. However, ECM and vitamin E treatment groups exhibited tightly packed and regularly arranged neurons (Supplementary Figure 1). In line with this, ECM or vitamin E treatment sustained the expression levels of PSD95 and SYN (Figures 5(d) and 5(e)). The results indicate that ECM could protect neurons against oxidative stress in a D-gal/AlCl_3_-induced neurodegenerative mouse model.

### 3.6. ECM Protects Hippocampus Neurons from Oxidative Stress via the Nrf2 Signaling Pathway

**Figure 6:**
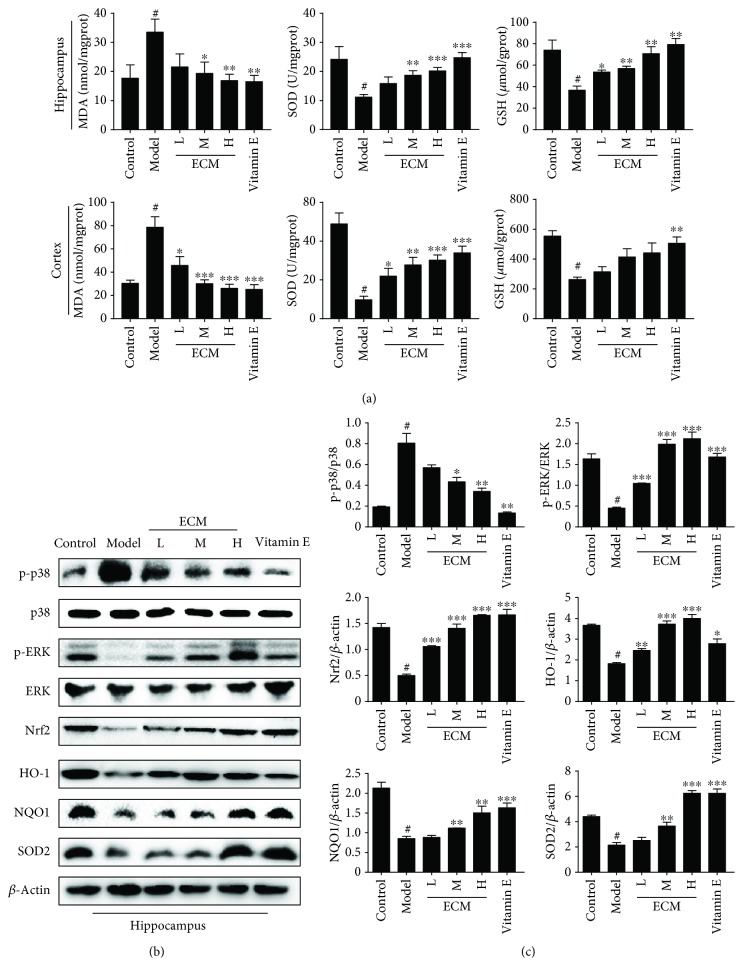
ECM inhibits oxidative stress via the MAPK and Nrf2 signaling pathways in the CNS. The homogenate of the hippocampus and cortex was used for the assay, and the lysate of the hippocampus was used for Western blot. (a) The content of MDA and the activities of SOD and GSH in the hippocampus (upper panel) and cortex (lower panel) were measured using a kit assay. (b) Western blot analysis of p-p38, p38, p-ERK, ERK, Nrf2, HO-1, NQO-1, and SOD2; *β*-actin was used as a loading control in the hippocampus. (c) Relative protein levels were quantified by densitometry and normalized to *β*-actin. Control: vehicle control; ECM-L: D − gal/AlCl_3_ + ECM (100 mg/kg/d); ECM-M: D − gal/AlCl_3_ + ECM (200 mg/kg/d); ECM − H : D − gal/AlCl_3_ + ECM (400 mg/kg/d); vitamin E: D − gal/AlCl_3_ + vitamin E (80 mg/kg/d). Data are represented as the mean ± SEM (*n* = 10 per group). ^#^ *p* < 0.05 and ^##^ *p* < 0.01 in comparison with the control group; ^∗^ *p* < 0.05, ^∗∗^ *p* < 0.01, and ^∗∗∗^ *p* < 0.001 in comparison with the model group.

To determine the antioxidant effects of ECM, we first detected the levels of MDA, GSH, and SOD activities in the brain. In the hippocampus and cortex of D-gal/AlCl_3_-treated mice, the level of MDA was significantly higher, and the SOD activity and GSH level were lower than those of the control group (Figure 6(a)); these levels were markedly reversed by ECM treatment. In particular, the levels in the high-dose ECM group were comparable to those in the vitamin E group (Figure 6(a)). These results indicate that ECM exerts neuroprotective effects via alleviation of oxidative stress in a D-gal/AlCl_3_ mouse model.

To determine whether ECM ameliorates oxidative stress via the MAPK and Nrf2 signaling pathways in D-gal/AlCl_3_-challgened mice, the levels of phosphorylated p38 MAPK and ERK and phase II detoxification enzymes in the hippocampus were examined by Western blot. In line with the *in vitro* results, phosphor-p38 MAPK was increased, while phosphor-ERK was decreased, in the hippocampus of D-gal/AlCl_3_-treated mice (Figures 6(b) and 6(c)). However, ECM and vitamin E treatment reduced the phosphorylation of p38 MAPK and increased the phosphorylation of ERK, especially in the high-dose ECM and vitamin E treatment groups (Figures 6(b) and 6(c)). We then detected the expression levels of Nrf2 and its downstream proteins. Nrf2, HO-1, SOD2, and NQO-1 had relatively lower expression levels in the D-gal/AlCl_3_ model group than those in the control group, whereas ECM or vitamin E treatment significantly increased the levels of Nrf2, HO-1, SOD2, and NQO-1 (Figures 6(b) and 6(c)), which was consistent with the *in vitro* results. These data suggest that ECM treatment can activate the Nrf2 signaling pathway and induce the expression of phase II detoxification enzymes, which are responsible for ameliorating oxidative stress and improving neurodegenerative diseases.

## 4. Discussion

Numerous reports have indicated that natural products harbor great chemical diversity, in turn exhibiting multiple biological activities. Increasing evidence has demonstrated that some natural products contain antioxidants that protect against oxidative stress in chronic disease, especially aging-related diseases. Identification of safe therapeutic regimens for oxidative stress-related diseases has attracted great attention. *C. minima* has been used as a medicinal herb to treat a number of diseases. Both aqueous and hydroalcoholic extracts of *C. minima* have been reported to scavenge free radicals and ameliorate oxidative stress, and the hydroalcoholic extracts express higher antioxidant activity [32]. However, the underlying mechanism of *C. minima* as an antioxidant agent was previously unknown. In this study, we examined the therapeutic potential and underlying mechanism of ECM in the treatment of oxidative stress-induced neurodegenerative disease and found that the ethanol extracts of *C. minima* exhibited significant antioxidant activity both *in vitro* and *in vivo*, mainly through upregulating nonenzymatic and enzymatic antioxidants. In this study, we also found that ECM protected mitochondrial function through the reduction of oxidative stress and by sustaining the Bcl-2/Bax ratio. Therefore, ECM could protect neurons in the brain from oxidative stress, which in turn attenuates the process of neurodegeneration and improves learning and memory capacity in a D-gal/AlCl_3_-induced mouse model.

Although polyphenol and flavonoids are thought to be responsible for the antioxidant activity of *C. minima*, the active compounds had not been identified. A series of sesquiterpene lactones have been isolated from *C. minima*, with 6-*O*-angeloylplenolin found to be one of the most abundant compounds in ECM; 6-*O*-angeloylplenolin has been reported to exhibit antiallergy, antibacterial, and antiproliferative effects [34–37]. Arnicolide D and arnicolide C have also been shown to have antibacterial and antiproliferative effects [28, 36]. However, the antioxidant effects of these sesquiterpenoids in *C. minima* have never been reported. Therefore, we examined whether the sesquiterpenoids mediated the antioxidant activity of ECM. Intriguingly, we found that all four sesquiterpenoids had antioxidant activity, as they inhibited the neuronal death induced by tBHP, with 6-*O*-angeloylplenolin and arnicolide D exhibiting antioxidant activity even at low concentrations. Meanwhile, 6-*O*-angeloylplenolin and arnicolide D markedly induced the expression of phase II detoxification enzymes responsible for alleviating oxidant stress. Moreover, we analyzed the concentration of the compounds in ECM and found that both 6-*O*-angeloylplenolin and arnicolide D were highly concentrated in the extract of *C. minima* (see Supplementary Material), further indicating that 6-*O*-angeloylplenolin and arnicolide D act as active compounds that exhibit antioxidant properties. Our results suggest that sesquiterpenoids could be considered the active compounds in *C. minima* responsible for ameliorating oxidative stress; therefore, the therapeutic potential of sesquiterpenoids in treating neurodegenerative diseases warrants further investigation. The compound 6-*O*-angeloylplenolin and arnicolide D have been reported to exhibit antiproliferative activities against cancer cells; however, no obvious cytotoxicity was observed in our study, which may be due to that the compounds were used at lower concentrations than that used for antiproliferation studies, indicating that the compounds exert different activities at different concentrations. We will further examine the toxic effects of 6-*O*-angeloylplenolin and arnicolide D *in vivo*.

Increasing evidence has demonstrated that the transcription factor Nrf2 plays a key role in antagonizing oxidative stress, with Nrf2 exerting cytoprotective effects via the upregulation of a series of phase II detoxification enzymes. Recently, certain Nrf2 inducers have been studied clinically, and Nrf2 has been considered an emerging therapeutic target [47]. Nrf2 activation is under strict regulation; usually, Nrf2 is retained in the cytoplasm by Kelch-like ECH-associated protein 1 (Keap1) and tends to be degraded. Certain kinases, including ERK, protein kinase C, and AKT, regulate Nrf2 activation, and multiple natural products have been reported to regulate Nrf2 activation and nuclear translocation. Vitamin E and sulforaphane have been extensively examined for the ability to attenuate oxidative stress via the regulation of the Nrf2 signaling pathway. In this study, we used vitamin E as a positive agent for attenuating oxidative stress and found that ECM ameliorated oxidative stress both *in vitro* and *in vivo*, mainly through the activation of ERK and induction of Nrf2 nuclear translocation. The active compounds 6-*O*-angeloylplenolin and arnicolide D also markedly induced activation of Nrf2. Our results suggest that the ECM-induced activation of Nrf2 may occur, at least in part, through the induction of ERK activation. It is also possible that the active compounds in ECM directly disrupt the binding interface between Nrf2 and Keaps. Therefore, the role of the active compounds of *C. minima* in Nrf2 activation requires further investigation.

## 5. Conclusion

In summary, we found that the ethanol extract of *C. minima* is able to protect neuronal cells against oxidative stress-induced neurodegeneration through activation of the Nrf2 signaling pathway. We also found that 6-*O*-angeloylplenolin and arnicolide D are the potential active compounds in *C. minima*, which provides strong evidence that *C. minima* has a therapeutic potential in ameliorating neurodegenerative diseases. *C. minima* could be used as a source of antioxidant and neuroprotective compounds.

## Data Availability

All data used to support the findings of this study are included within the article.

## Abbreviations

ECM: Ethanol extract of *C. minima*
tBHP: tert-Butyl hydroperoxide
D-gal: D-Galactose
AlCl_3_: Aluminum chloride
ROS: Reactive oxygen species
JNK: c-Jun N-terminal kinases
ERK: Extracellular signal-regulated kinases
MDA: Malonyl dialdehyde
GST: Glutathione S-transferase
MAPK: Mitogen-activated protein kinase
Nrf2: Nuclear factor erythroid 2-related factor 2
HO-1: Heme oxygenase-1
NQO-1: NAD(P)H: quinone oxidoreductase-1
SOD: Superoxide dismutase
GSH: Glutathione
PSD95: Postsynaptic density protein 95
SYN: Synaptophysin
HPLC: High-performance liquid chromatography
DCFH-DA: 2′,7′-Dichlorofluorescin diacetate.

## Supplementary Materials

Supplementary Materials Material and Method for extraction and isolation of EBSC-26A–EBSC-26D and 9 figures. Supplementary Figure 1: ECM exerts neuroprotective effects in the hippocampus. Supplementary Figure 2–9: 1H NMR and 13C NMR spectra of EBSC-26A–EBSC-26D.
