# Supplementary material for: Ethanol Extract of Centipeda minima Exerts Antioxidant and Neuroprotective Effects via Activation of the Nrf2 Signaling Pathway
Source: Oxid Med Cell Longev. 2019 Apr 3;2019:9421037. doi: 10.1155/2019/9421037 (PMC6470452; doi:10.1155/2019/9421037)
Supplement: Supplementary Materials — Material and Method for extraction and isolation of EBSC-26A–EBSC-26D and 9 figures. Supplementary Figure 1: ECM exerts neuroprotective effects in the hippocampus. Supplementary Figure 2–9: 1H NMR and 13C NMR spectra of EBSC-26A–EBSC-26D. [file 9421037.f1.pdf]

Supplementary Materials

Ethanol extract of *Centipeda minima* exerts antioxidant and neuroprotective effects via activation of Nrf2 signaling

Yi-Jie Wang[1#], Xin-Yue Wang[3#], Xu-Yi Hao[1], Yong-Ming Yan[2], Ming Hong[1], Su-Fen Wei[1], Yi-Le Zhou[1], Qi Wang[1]*, Yong-Xian Cheng[2]*, Yong-Qiang Liu[1]*

[1]Institute of Clinical Pharmacology, Guangzhou University of Chinese Medicine, Guangzhou 510405, PR China

[2]School of Pharmaceutical Sciences, Shenzhen University Health Science Center, Shenzhen 518060, PR China

[3]Sun Yat-sen University Cancer Center, Guangzhou 510060, PR China

[#] These authors have contributed equally to this work

*Correspondence should be addressed to Yong-Qiang Liu; liuyq@gzucm.edu.cn; Yong-Xian Cheng: yxcheng@szu.edu.cn; Qi Wang; wangqi@gzucm.edu.cn.

Supplementary Materials and Methods

Hematoxylin-eosin (H&E) staining

At the end of animal experiments, mice were anesthetized and perfused with ice-cold PBS and 4% paraformaldehyde. In brief, sagittal sections at 10 μm were prepared by using a Leica ultra-thin semiautomatic microtome as previously described after tissue dehydration. Brain paraffin sections were washed in xylene and rehydrated through a graded series of ethanol and double-distilled water. Then the sections were dipped in hematoxylin staining (Beyotime, Shanghai, China) for 10 min and eosin staining (Beyotime, Shanghai, China) for 1 min at room temperature. Then slides were rinsed in double-distilled water and dehydrated through 80%, 90% and 100% alcohol, cleared in xylene. Images were analyzed by using a light microscope and LEICA QWin Plus (Leica Microsystems, Wetzlar, Germany).

Extraction and isolation of EBSC-26A–EBSC-26D

The air-dried and powdered *Centipeda minima* whole plants (60 kg) were extracted by refluxing with 70% EtOH (3 × 120 L × 2 h) to give a crude extract, which was suspended in water followed by extraction with EtOAc to afford an EtOAc soluble extract. The EtOAc extract was suspended in petroleum ether/MeOH (1:1) to afford the MeOH extract. The MeOH partition was applied to a silica gel (200-300 mesh) column, eluted with petroleum ether-EtOAc (10:1-0:1 gradient system), to give fractions A to F. Fraction B was divided into eight portions (B1–B8) by MCI gel CHP 20P eluted with gradient aqueous MeOH

(30%–70%). Fraction B6 was subjected to a RP-18 column (MeOH/H_2O, 40%–70%) to provide six portions (B6.1–B6.6) monitored by TLC using previously purified EBSC-26A as a reference compound. Fraction B6.3 was recrystallized to afford EBSC-26A (30.0 g, content 2.5‰). Fraction B6.4 was further purified using preparative HPLC (MeOH/H_2O, 65%) to yield EBSC-26A (3.0 g), EBSC-26B (2.0 g, content 0.7‰), EBSC-26C (0.8 g), and EBSC-26D (0.6 g).

EBSC-26A, white solids, ESIMS: *m/z* 369 $[M + Na]^+$; ^{1}H NMR (600 MHz, $CDCl_3$), see Supplementary Figure 2; ^{13}C NMR (150 MHz, $CDCl_3$), see Supplementary Figure 3.

EBSC-26B, white solids, ESIMS: *m/z* 355 $[M + Na]^+$; ^{1}H NMR (600 MHz, $CDCl_3$), see Supplementary Figure 4; ^{13}C NMR (150 MHz, $CDCl_3$), see Supplementary Figure 5.

EBSC-26C, white solids, ESIMS: *m/z* 357 $[M + Na]^+$; ^{1}H NMR (600 MHz, $CDCl_3$), see Supplementary Figure 6; ^{13}C NMR (150 MHz, $CDCl_3$), see Supplementary Figure 7.

EBSC-26D, white solids, ESIMS: *m/z* 369 $[M + Na]^+$; ^{1}H NMR (600 MHz, $CDCl_3$), see Supplementary Figure 8; ^{13}C NMR (150 MHz, $CDCl_3$), see Supplementary Figure 9.

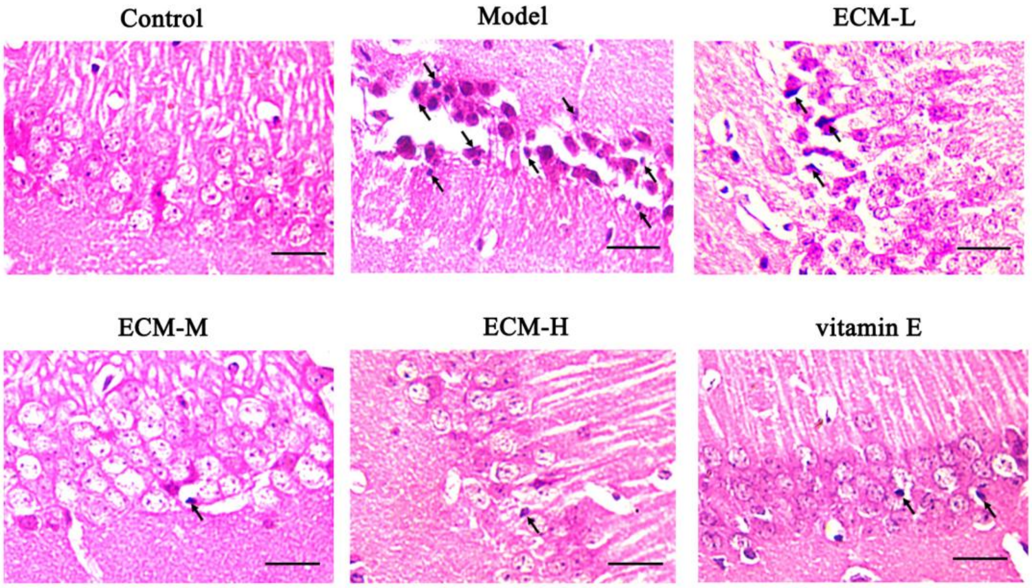

Supplementary Figure 1. ECM exerts neuroprotective effects in the hippocampus. H&E staining show morphological changes of neurons in the hippocampus. Scale bar, 25 μm. Control: vehicle control; ECM-L: D-gal/$AlCl_3$ + ECM (100 mg/kg/d); ECM-M: D-gal/$AlCl_3$ + ECM (200 mg/kg/d); ECM-H: D-gal/$AlCl_3$ + ECM (400 mg/kg/d); vitamin E: D-gal/$AlCl_3$ + vitamin E (80 mg/kg/d).

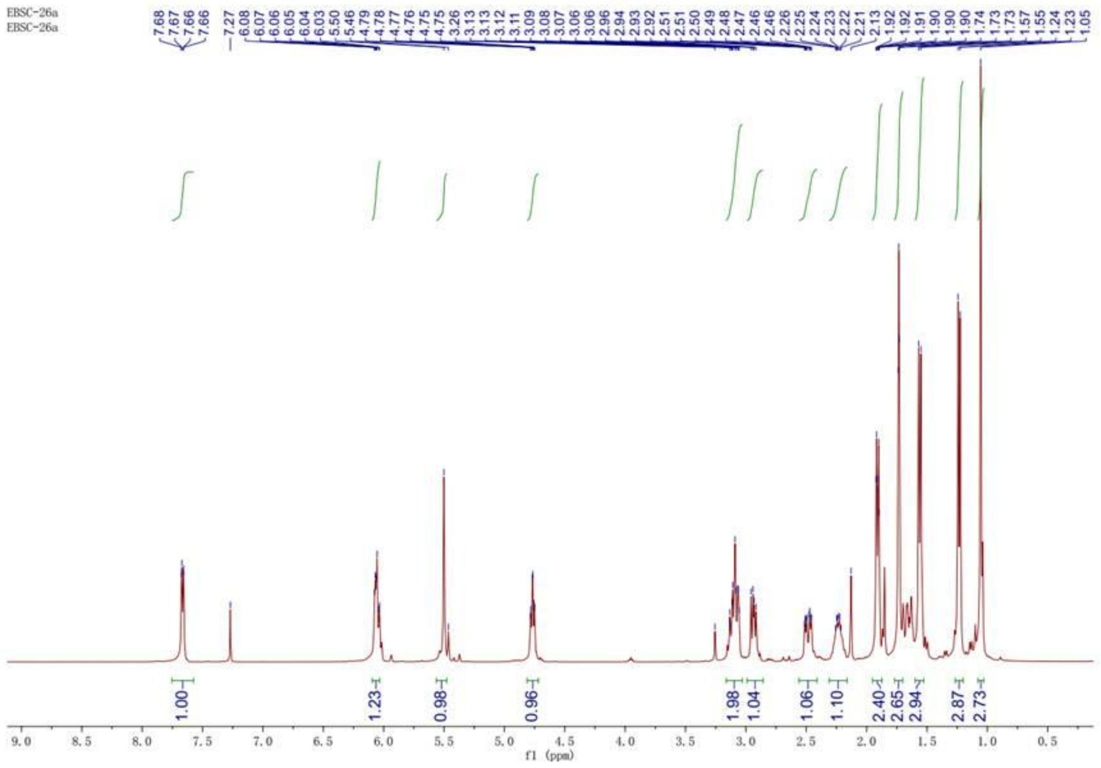

Supplementary Figure 2. ^{1}H NMR spectrum of **EBSC-26A** in $CDCl_3$.

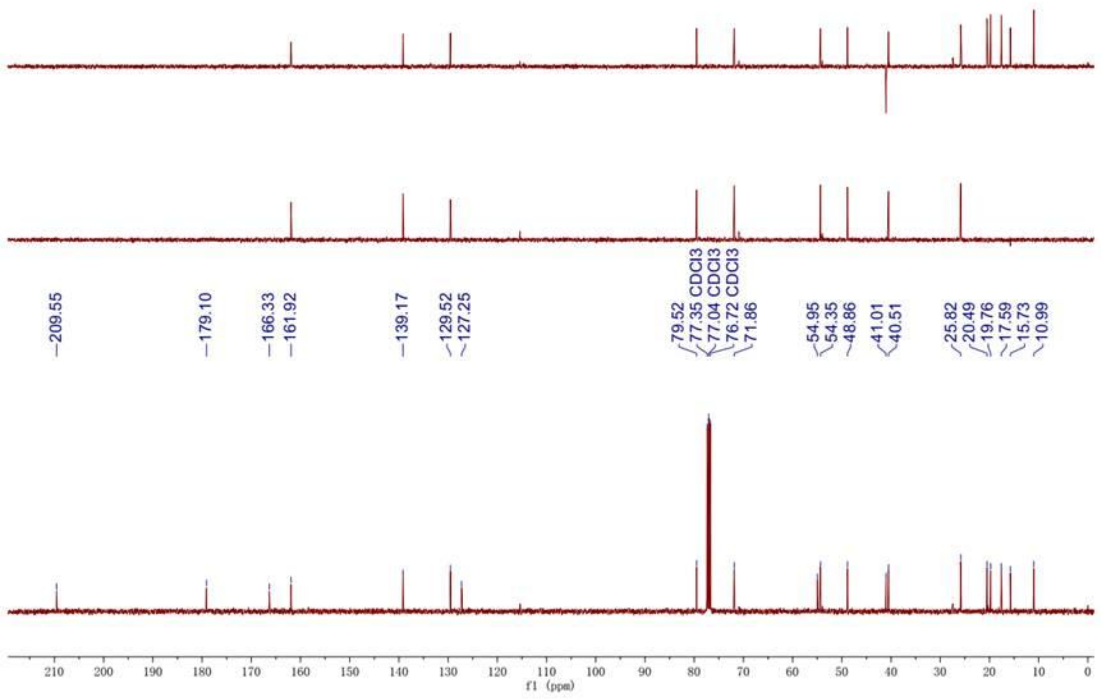

Supplementary Figure 3. ^{13}C NMR and DEPT spectra of **EBSC-26A** in $CDCl_3$.

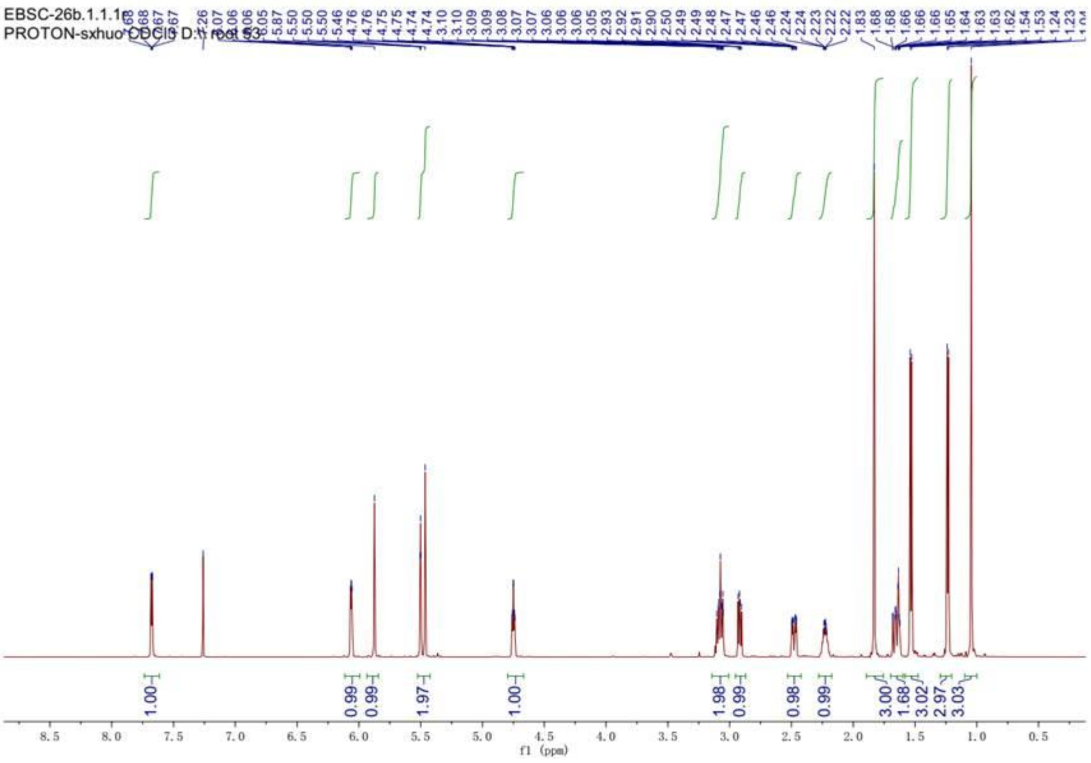

Supplementary Figure 4. ^{1}H NMR spectrum of **EBSC-26B** in $CDCl_{3.}$

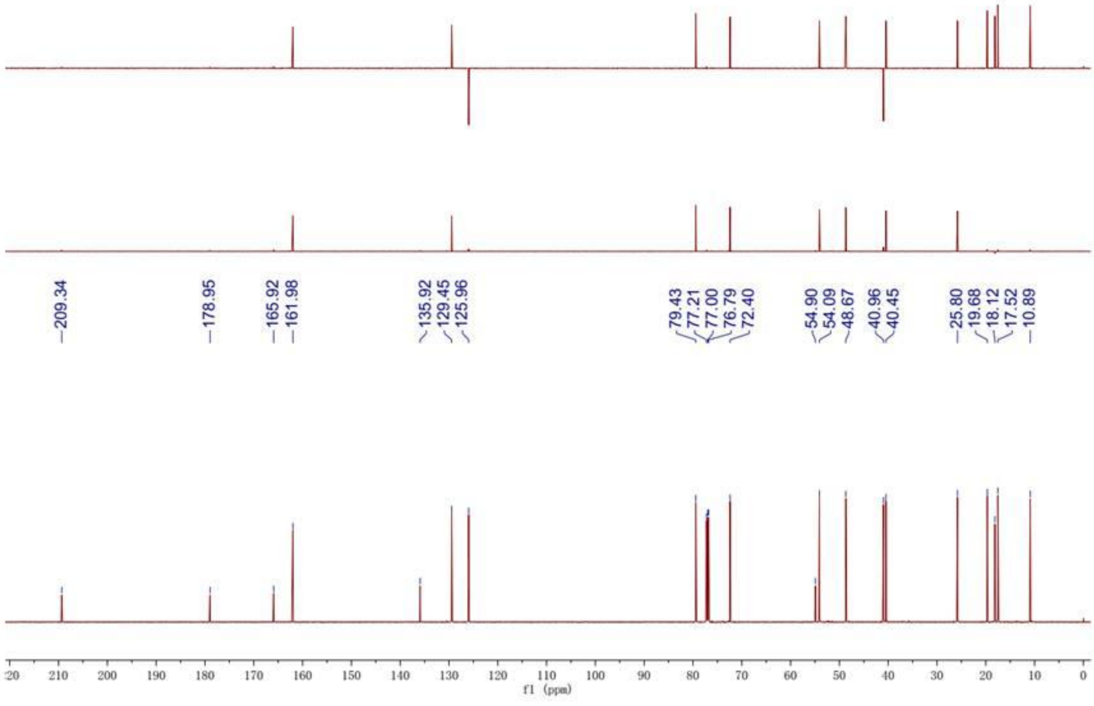

Supplementary Figure 5. ^{13}C NMR and DEPT spectra of **EBSC-26B** in $CDCl_{3.}$

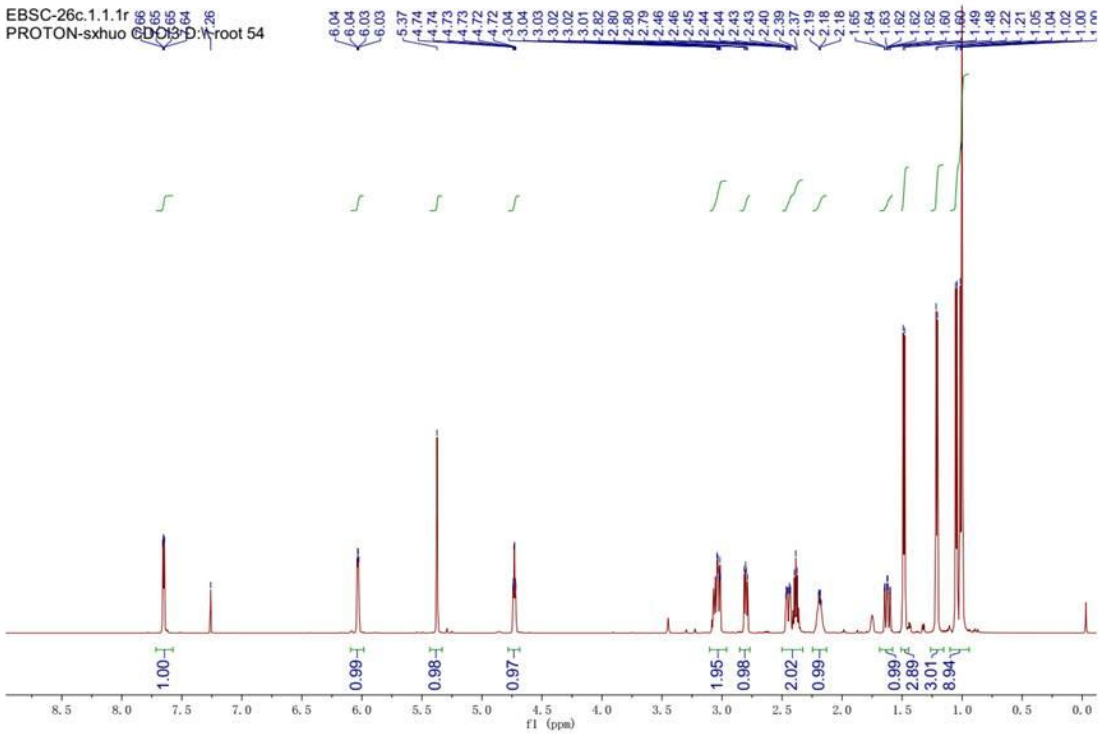

Supplementary Figure 6. ^{1}H NMR spectrum of **EBSC-26C** in $CDCl_3$.

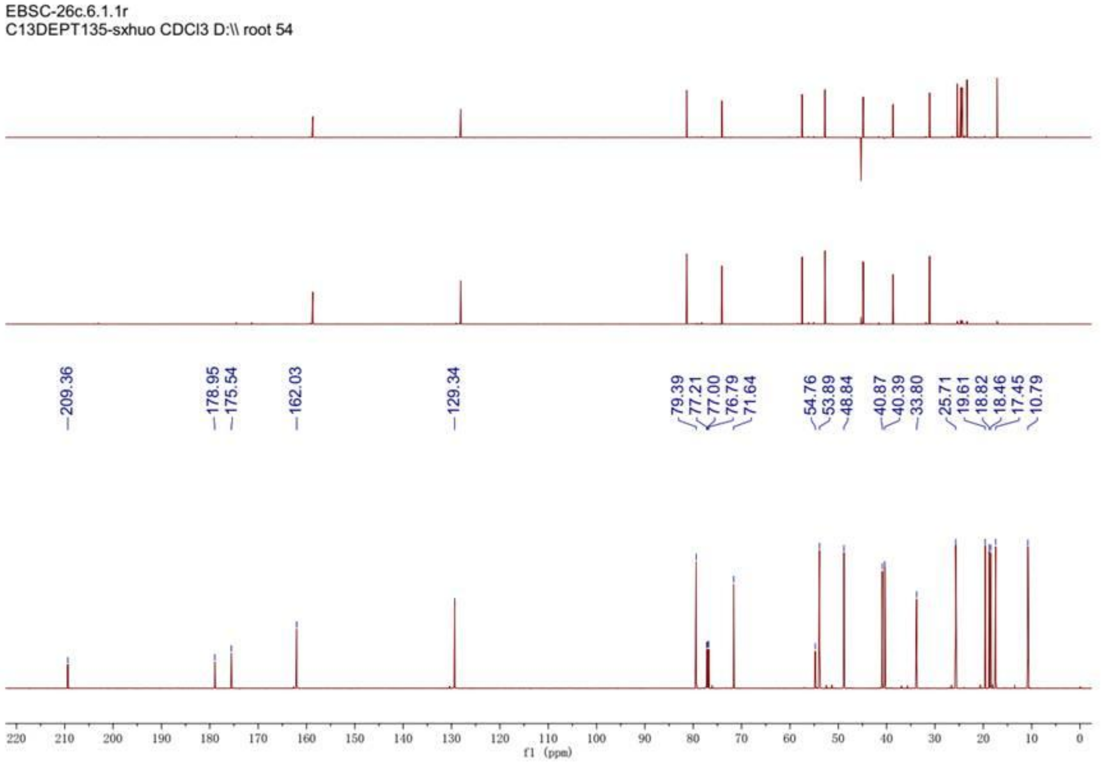

Supplementary Figure 7. ^{13}C NMR and DEPT spectra of **EBSC-26C** in $CDCl_3$.

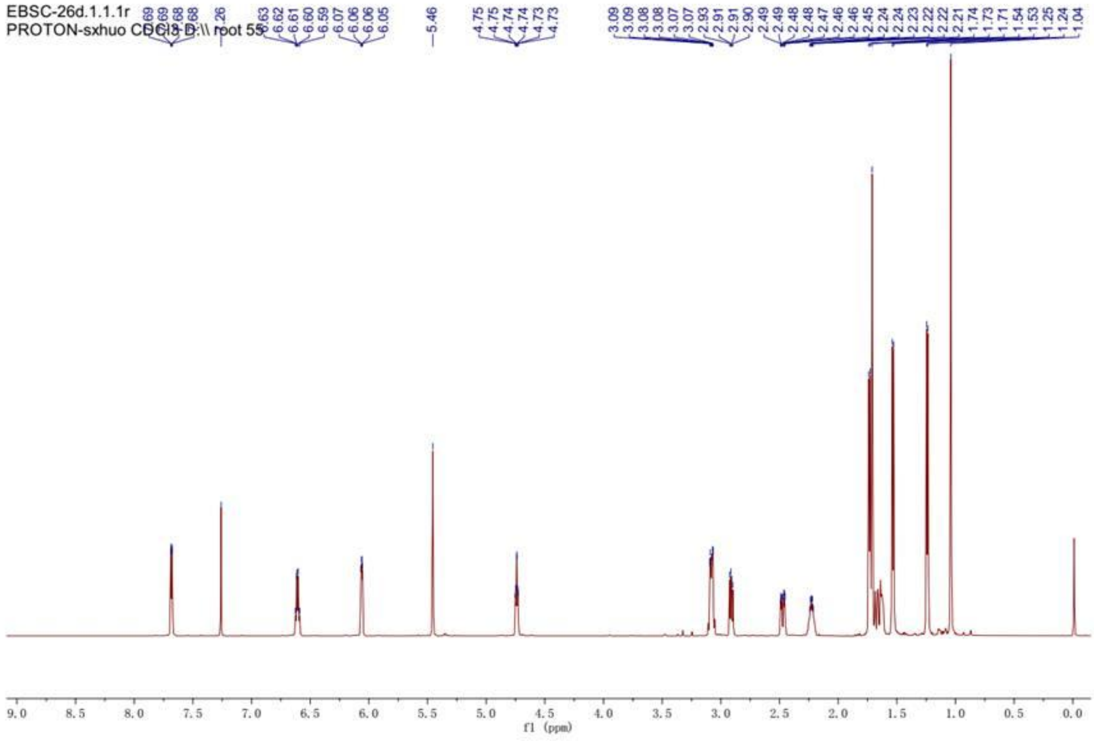

Supplementary Figure 8. ^{1}H NMR spectrum of **EBSC-26D** in $CDCl_{3.}$

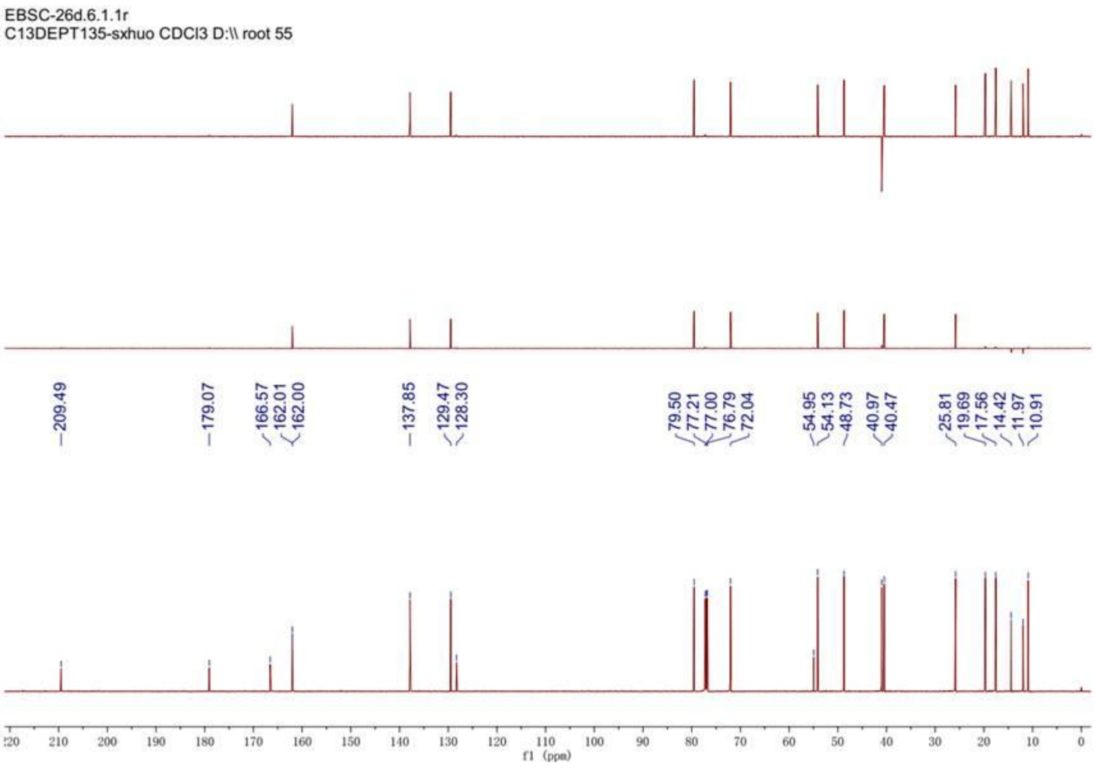

Supplementary Figure 9. ^{13}C NMR and DEPT spectra of **EBSC-26D** in $CDCl_{3.}$
